# SRSF7 downregulation induces cellular senescence through generation of *MDM2* variants

**DOI:** 10.18632/aging.205420

**Published:** 2023-12-29

**Authors:** Jiwon Hong, Seongki Min, Gyesoon Yoon, Su Bin Lim

**Affiliations:** 1 Department of Biochemistry and Molecular Biology, Ajou University School of Medicine, Suwon 16499, Korea; 2 Inflamm-aging Translational Research Center, Ajou University Medical Center, Suwon 16499, Korea; 3 Department of Biomedical Sciences, Graduate School of Ajou University, Suwon 16499, Korea

**Keywords:** senescence, cellular senescence, aging, alternative splicing, mouse double minute 2 homolog (MDM2)

## Abstract

Alternative splicing (AS) enables a pre-mRNA to generate different functional protein variants. The change in AS has been reported as an emerging contributor to cellular senescence and aging. However, it remains to be elucidated which senescent AS variants are generated in and regulate senescence. Here, we observed commonly down-regulated SRSF7 in senescent cells, using publicly available RNA-seq datasets of several *in vitro* senescence models. We further confirmed SRSF7 deregulation from our previous microarray datasets of time-series replicative senescence (RS) and oxidative stress-induced senescence (OSIS) of human diploid fibroblast (HDF). We validated the time-course changes of SRSF mRNA and protein levels, developing both RS and OSIS. SRSF knockdown in HDF was enough to induce senescence, accompanied by p53 protein stabilization and MDM2 variants formation. Interestingly, expression of MDM2 variants showed similar patterns of p53 expression in both RS and OSIS. Next, we identified MDM2-C as a key functional AS variant generated specifically by SRSF7 depletion. Finally, we validated that MDM2-C overexpression induced senescence of HDF. These results indicate that SRSF7 down-regulation plays a key role in p53-mediated senescence by regulating AS of MDM2, a key negative regulator of p53, implying its critical involvement in the entry into cell senescence.

## INTRODUCTION

Alternative splicing (AS) is a process that enables a pre-mRNA to synthesize different protein variants that may have different cellular functions or properties. Approximately 95% of human genes undergo alternative splicing, which results in the expansion of transcriptome and proteome diversity [1–3]. The AS process takes multiple roles in the regulation of various biological processes, including cell and tissue homeostasis [4] and organ development [5]. It has further emerged as an important contributor to aging and cellular senescence [6–8], as well as aging-associated diseases [9], including cardiovascular diseases [7] and cancer [10–12]. Particularly, it has been suggested that splicing deregulation caused by changes in the expression of splicing factors (SFs) may be involved in aging and senescence [13–15]. The altered expression of SFs might lead to increased AS variants of their target genes, including senescence-associated genes. For example, the depletion of splicing factor PRPF19 induced senescence in human diploid fibroblasts by altering the splicing patterns of MDM4 [16]. Altogether, these studies suggest that proper regulation of AS process accompanied by balanced expression of SFs is important to inhibit senescence and aging. Nevertheless, it remains to be elucidated how specific SFs could regulate the AS of target genes and how their depletion could induce cellular senescence.

There are multiple types of AS. The major types include exon skipping, alternative 3′ splice sites, alternative 5′ splice sites, mutually exclusive exons and intron retention. Among these different types, exon skipping is the most common type of senescence-associated differential splicing events [15]. Splicing process is regulated by various splicing regulators, including SFs, which interact with spliceosome and help to recognize splice sites in pre-mRNA [17]. There are two major SF families, serine and arginine rich splicing factor (SRSF) family and heterogeneous nuclear ribonucleoprotein (hnRNP) family. SRSF proteins mainly recognize exonic splicing enhancer sequences and promote the inclusion of those recognized exons, while hnRNPs mainly recognize exonic splicing silencer sequences and repress exon inclusion [18, 19]. Considering that the exon skipping is the most common type of senescence-associated differential splicing events, the proper regulation in exon recognition and subsequent exon inclusion is critical to control senescence. In particular, SRSF proteins have been inextricably linked to cellular senescence and aging-associated phenotypes [7, 20–23]. For example, SRSF1 and SRSF7 have been identified as key splicing regulatory RNA binding proteins in senescence through systemic analyses of transcriptomic data [15]. Moreover, it has been recently proposed that juvenile-expressed SRSF7 may mediate the age-dependent AS and its depletion may lead to growth cessation in mice [22]. These findings suggest that SRSF7 is essential for cell growth and its depletion can lead to senescence. Despite its apparent relevance to the cell growth in cellular senescence, molecular basis of SRSF7 depletion mediating senescence remains unclear.

Tumor suppressor p53 is known to regulate senescence, especially through the induction of its downstream effector p21 [24, 25]. The stability of p53 protein is negatively regulated by mouse double minute 2 homolog (MDM2) [26–28]. MDM2 binds to p53 and ubiquitinates it, leading to the proteasomal degradation of p53. The disruption of the MDM2-p53 interaction leads to the accumulation of p53 in cells [29], suggesting that the maintenance of balanced regulation between MDM2 and p53 is important to prevent p53-mediated senescence. Interestingly, MDM2 has various splice isoforms [30]. Many MDM2 splice isoforms lack functional domains, including p53 binding domain [31], suggesting that they may lose the ability to bind and degrade p53. Several studies have provided molecular evidence that the presence of MDM2 splice isoforms, which lack p53 binding domain but retain RING domain, can bind to MDM2 full-length (MDM2-FL) and inhibit its function in p53 degradation [32–35], thereby inducing p53 activation and subsequent cell cycle arrest [36–38]. Despite the evident role of MDM2 splice variants in senescence, however, it remains to be investigated how these variants are generated during senescence and contribute to senescence.

Here, we investigated the role of SRSF7 in the regulation of AS of MDM2 during cellular senescence. Down-regulation of SRSF7 was observed during replicative senescence (RS) and oxidative stress-induced senescence (OSIS) in human diploid fibroblasts (HDFs), accompanied with increased generation of MDM2 splice variants. SRSF7 depletion led to the formation of various MDM2 splice isoforms, including MDM2-C. MDM2-C, whose exon 4–8 are skipped, lacks p53 binding domain but retains RING domain. MDM2-C expression in young fibroblasts was shown to induce cellular senescence. These results suggest that the proper splicing regulation of MDM2 by SRSF7 is crucial to prevent senescence, implying that balanced AS regulation may play an important role in senescence.

## RESULTS

### SRSF7 is commonly down-regulated in senescent cells

**Figure 1:**
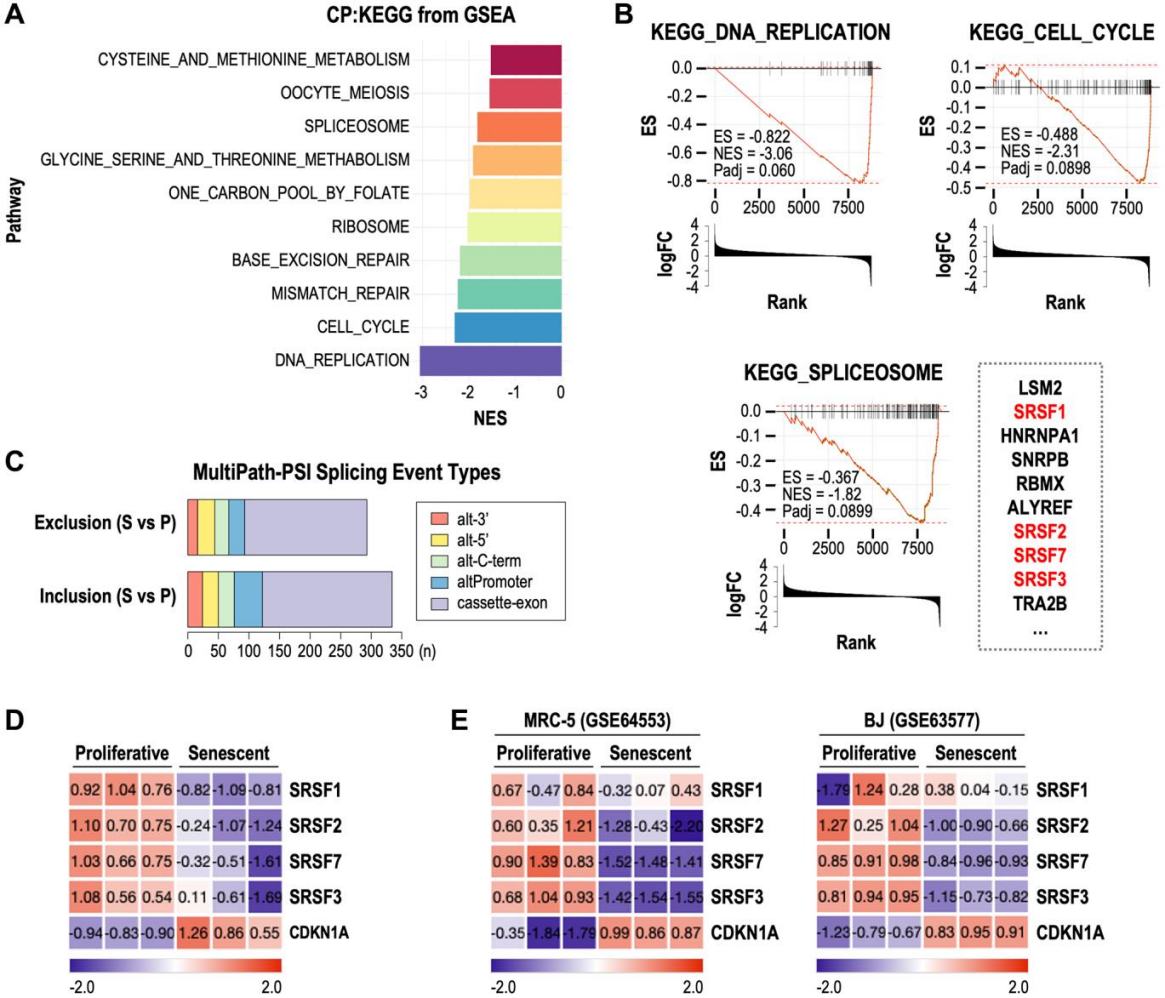
**Down-regulation of SRSF7 is commonly observed in multiple RNA-seq datasets of cellular senescence.** (**A**–**D**) RNA seq analysis of Wi-38 cells during RS (GSE130306). (**A**) Bar plot showing the top gene sets which were downregulated in senescence cells. (**B**) GSEA plot showing the enrichment score (ES) and normalized enrichment score (NES) and adjusted *p*-value (*P*adj) of three gene sets (DNA_REPLICATION, CELL_CYCLE, and SPLICEOSOME) in senescent cells. Bar plots showing the log2 fold change (logFC) were displayed together. Top ten leading edge genes in ‘SPLICEOSOME’ gene set are shown next to its GSEA plot. (**C**) Stacked bar plot showing the number of differential splicing event types in senescent (S) cells compared to proliferative (P) cells. Abbreviations: alt-3’: alternative 3’ splice sites; alt-5: alternative 5’ splice sites; alt-C-term: alternative C terminal exons; altPromoter: alternative promoters; cassette-exon: alternative exon-cassettes. (**D**) Gene expression heatmap of CDKN1A and SRSF genes included in the leading edge genes of ‘SPLICEOSOME’ gene set. The values are scaled into z-score. (**E**) Gene expression heatmaps of CDKN1A and SRSF genes from RNA seq data of MRC-5 (GSE64553) and BJ (GSE63577) during RS. The values are scaled into z-score.

To investigate how splicing deregulation is involved in cellular senescence, we performed gene set enrichment analysis (GSEA) using publicly available RNA-seq data derived from RS model of human fibroblasts (see Methods). Compared to proliferative fibroblasts, in senescent fibroblasts, ‘DNA_REPLICATION’ and ‘CELL_CYCLE’ gene sets were most down-regulated (Figure 1A, 1B). Interestingly, ‘SPLICEOSOME’ gene set was also significantly down-regulated in senescent samples (Figure 1A, 1B). Through AltAnalyze analyses (see Methods), we found a total of 627 differential splicing events between proliferative and senescent fibroblasts (Figure 1C). Notably, most of them were exclusion and inclusion events of exons rather than of alternative 3′ or 5′ splice sites, alternative C terminal exons, alternative promoters. This result implies that the senescent spliceosomal deregulation generates diverse AS variants which were formed by exon exclusion and inclusion, thereby modulating senescence phenotypes. In addition, we identified that 4 SRSF family genes, SRSF1, SRSF2, SRSF7 and SRSF3, were among the top 10 ‘leading edge’ genes of ‘SPLICEOSOME’ gene set (Figure 1B). Given that the SRSF proteins are known to promote exon recognition for exon inclusion during splicing process [18], our findings suggest that SRSF deregulation-mediated AS events may closely be related to cellular senescence. Individual expression profiles of the four SRSFs showed clear down-expression in senescent samples of the RS model, accompanied with induction of cell cycle inhibitor, CDKN1A, known as a senescence marker gene (Figure 1D). Among the four down-regulated SRSF family genes, we focused on SRSF1 and SRSF7, which have previously been suggested to be key splicing regulatory RNA binding proteins in cellular senescence [15]. Unlike SRSF7, however, SRSF1 did not exhibit consistent down-expression in RS of MRC-5 and BJ cells (Figure 1E). These observations altogether suggest that SRSF7 down-expression plays a role in AS-modulated cellular senescence.

### SRSF7 depletion induces cellular senescence

**Figure 2:**
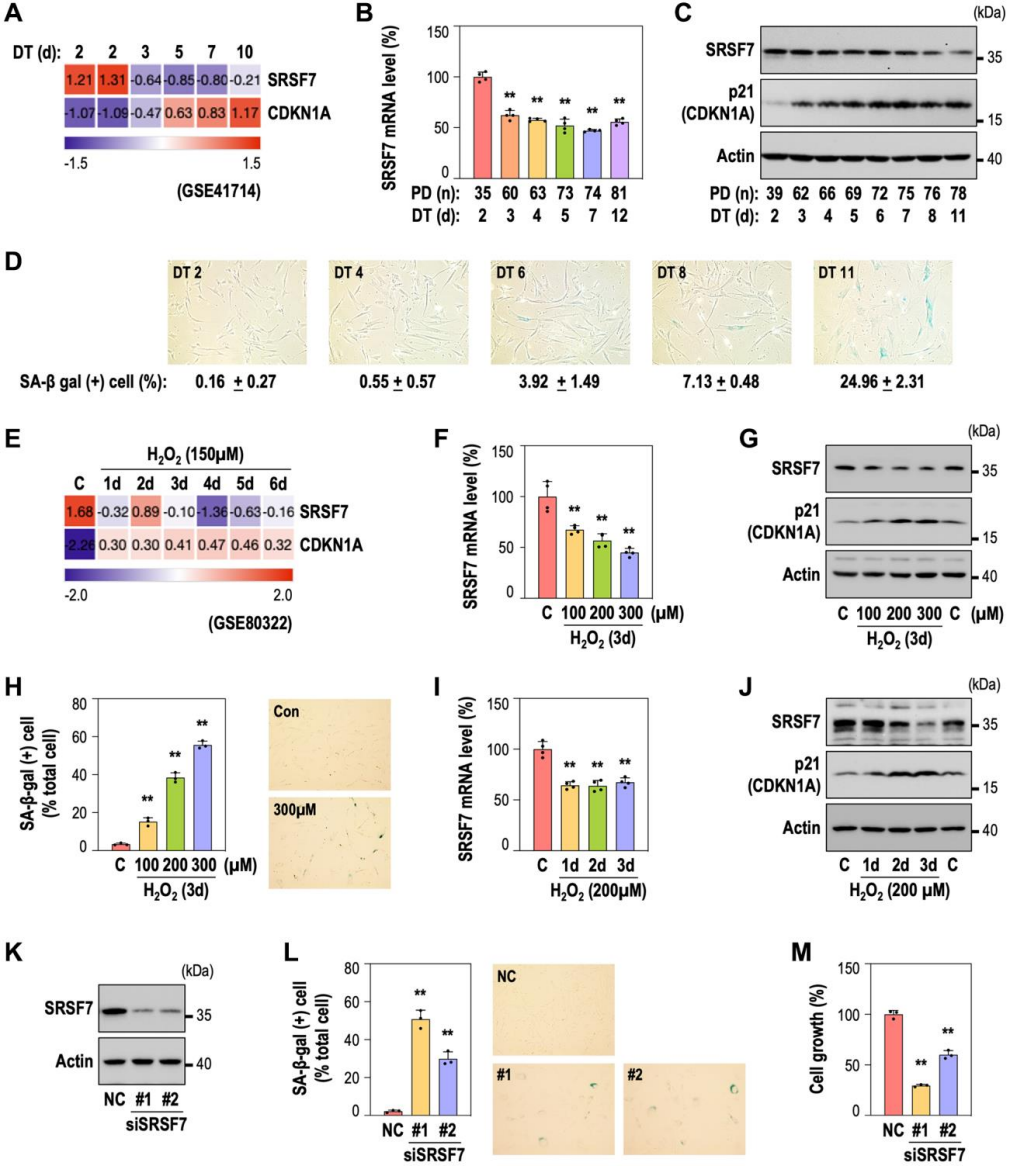
**SRSF7 depletion is involved in diverse models of cellular senescence.** (**A**–**D**) Expression of SRSF7 and senescence markers (CDKN1A, or p21 as its protein name, and SA-β gal activity) in RS model of HDFs. (**A**) Expression heatmap from publicly available microarray data (GSE41714). The values are scaled into z-score. (**B**) mRNA level of SRSF7 using qPCR. (^**^p < 0.01 vs. DT2 by student *t*-test). (**C**) Western blot analysis. (**D**) The percentage of SA-β-gal positive (+) cells along with pictures of stained cells. (**E**–**J**) Expression of SRSF7 and the same senescence markers in OSIS model of HDFs. (**E**) Expression heatmap from publicly available microarray data (GSE80322). The values are scaled into z-score. (**F**) mRNA level of SRSF7 in dose-dependent OSIS using qPCR. (^**^p < 0.01 vs. Con by student *t*-test). (**G**) Western blot analysis in dose-dependent OSIS. (**H**) The quantification of SA-β-gal (+) cells along with pictures of stained cells. (^**^p < 0.01 vs. Con by student *t*-test). (**I**) mRNA level of SRSF7 in time-dependent OSIS using qPCR. (^**^p < 0.01 vs. Con by student *t*-test). (**J**) Western blot analysis in time-dependent OSIS. (**K**–**M**) HDFs (DT2) were transfected with siRNA against either negative control (NC) or SRSF7 for 4 days. (**K**) Western blot analysis. (**L**) The quantification of SA-β-gal (+) cells along with pictures of stained cells. (^**^p < 0.01 vs. NC by student *t*-test). (**M**) The quantification of cell growth activity. (^**^p < 0.01 vs. NC by student *t*-test).

We previously reported the time-series gene expression profiles of RS and OSIS of HDFs [39, 40]. By employing the RS dataset (GSE41714), we also identified down-regulation of SRSF7 from the early time point (DT3), as shown in Figure 2A. By developing RS model of HDFs, we further validated SRSF7 mRNA and protein levels, together with CDKN1A (p21) expression and gain of senescence-associated beta galactosidase activity (SA-β-gal) (Figure 2B–2D). Interestingly, SRSF7 down-expression preceded the acquisition of SA-β-gal, the typical senescence phenotype. In the OSIS data set (GSE80322), SRSF7 down-regulation was similarly observed (Figure 2E). In addition, SRSF7 protein and mRNA levels showed obvious down-expression in dose-dependent manner, similarly to the gains of p21 and SA-β-gal, in OSIS model of HDFs (Figure 2F–2H). In time-course OSIS model, SRSF mRNA decreased from day 1, showing initial response but its protein expression showed time-dependent manner (Figure 2I, 2J). Next, to examine the direct link of SRSF7 depletion to cellular senescence, SRSF7 was suppressed by siRNA-mediated knockdown in primary HDF (Figure 2K). Notably, SRSF7 depletion significantly induced senescence, evidenced by SA-β-gal gain and cell growth inhibition (Figure 2L, 2M), suggesting that SRSF7 depletion could lead to cellular senescence.

### SRSF7 depletion-mediated senescence is related to the formation of MDM2 splice variants

**Figure 3:**
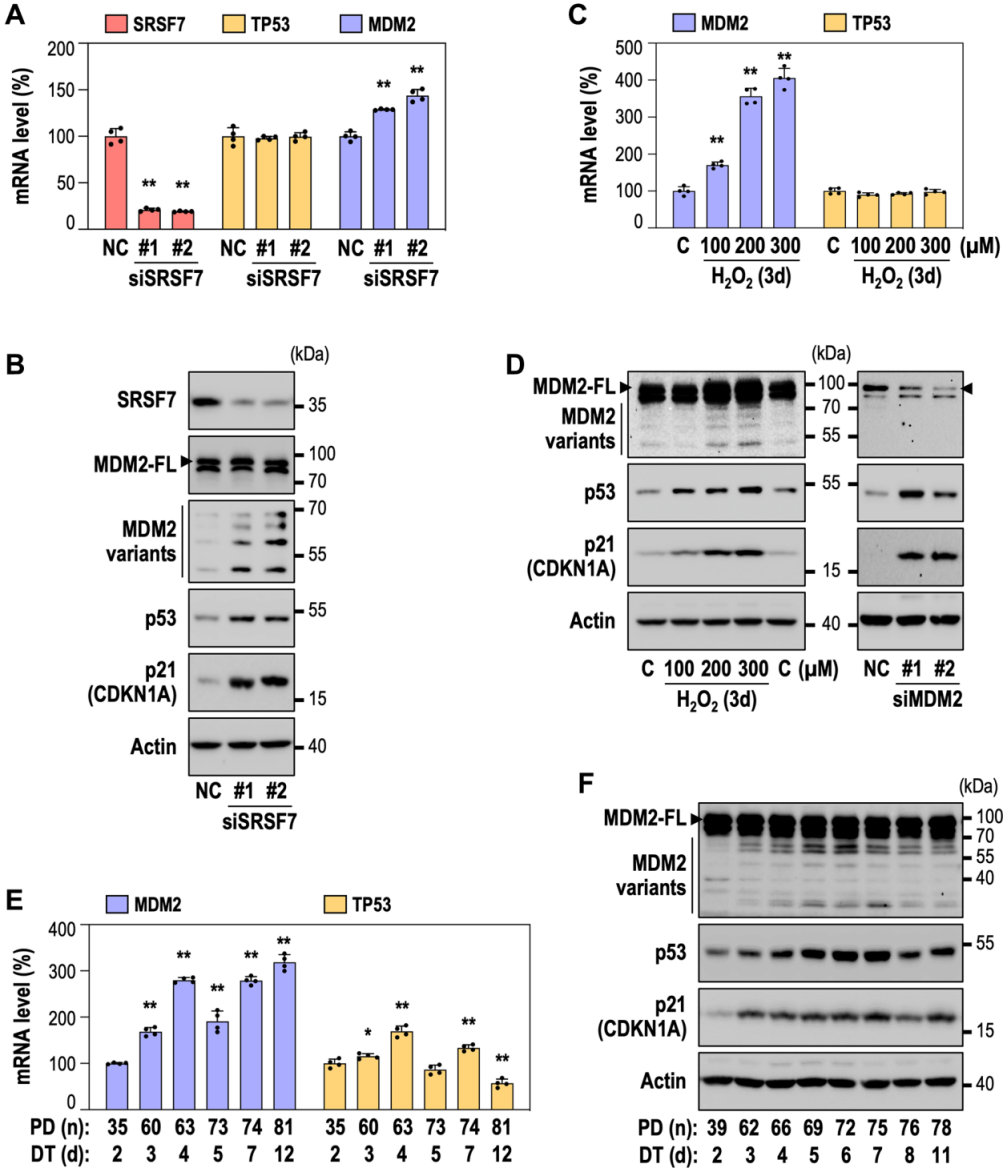
**SRSF7 knockdown-mediated senescence is accompanied by the generation of MDM2 splice variants.** (**A**, **B**) HDFs (DT2) were transfected with siRNA against either negative control (NC) or SRSF7 for 4 days. (**A**) mRNA level of SRSF7, TP53, MDM2 using qPCR. (^**^p < 0.01 vs. NC by student *t*-test). (**B**) Western blot analysis of MDM2 splice variants, p53 and p21. (**C**) mRNA level of MDM2 and TP53 in dose-dependent OSIS using qPCR. (^**^p < 0.01 vs. Con by student *t*-test). (**D**) Western blot analysis of MDM2 splice variants, p53 and p21 in dose dependent OSIS, along with western blot analysis of MDM2 knockdown. HDFs (DT2) were transfected with siRNA against either NC or MDM2. (**E**) mRNA level of MDM2 and TP53 in RS using qPCR. (^*^p < 0.05 and ^**^p < 0.01 vs. DT2 by student *t*-test). (**F**) Western blot analysis of MDM2 splice variants, p53 and p21 in RS.

Next, to elucidate how SRSF7 depletion could lead to cellular senescence, we examined MDM2-p53 axis, because the alterations in AS of MDM2 have been reported in cell cycle arrest by accumulation of p53, a upstream transcription regulator of p21 [36–38]. When SRSF7 was suppressed, TP53 mRNA level remained unchanged (Figure 3A) while protein expressions of p53 and p21, the downstream target of p53, were obviously increased (Figure 3B), suggesting that increase in p53 protein after SRSF7 knockdown is derived from increased p53 protein stability, regardless of TP53 mRNA level. Interestingly, the p53 protein induction was accompanied by total mRNA level of MDM2 (Figure 3A) and increased formation of MDM2 variants, along with slight increase of full-length form of MDM2 (Figure 3B). We further examined the formation of MDM2 splice variants in the two senescence models, RS and OSIS of HDF. In OSIS, total MDM2 mRNA was significantly increased, but TP53 mRNA unchanged (Figure 3C). At the protein level, MDM2 variants increased, along with induction of full-length MDM2 protein (MDM2-FL), p53 and p21 (Figure 3D). Interestingly, the formation of MDM2 splice variant was increasingly observed with H_2_O_2_ treatment (Figure 3D). The MDM2-FL was confirmed by siRNA-mediated knockdown of MDM2. Similarly, in the RS model of HDF, the total MDM2 mRNA was substantially increased (Figure 3E). Unlike OSIS model, however, TP53 did not show consistent mRNA level during RS, suggesting that this long-term senescence model might have diverse cellular events regulating TP53 mRNA (Figure 3E). Nevertheless, it is noteworthy that the formation of MDM2 variants were clearly observed in RS, accompanied with p53 and p21 induction (Figure 3F). These results show that SRSF7 depletion-mediated senescence may be closely related to the formation of MDM2 splice variants, which play a key role in p53 stabilization. Given that MDM2 protein is a E3 ligase to promote ubiquitin-mediated protein degradation of p53 [27, 28], high expression of MDM2 and low level of p53 protein in young HDF (DT2) seemed appropriate. However, it is difficult to explain how p53 protein increases in the presence of MDM2-FL. Our results suggest that MDM2 variants formed by SRSF7 down-regulation may block the action of MDM2-FL, consequently stabilizing p53 protein and inducing senescence.

### MDM2-C, a MDM2 AS variant, generated by SRSF7 depletion induces senescence

**Figure 4:**
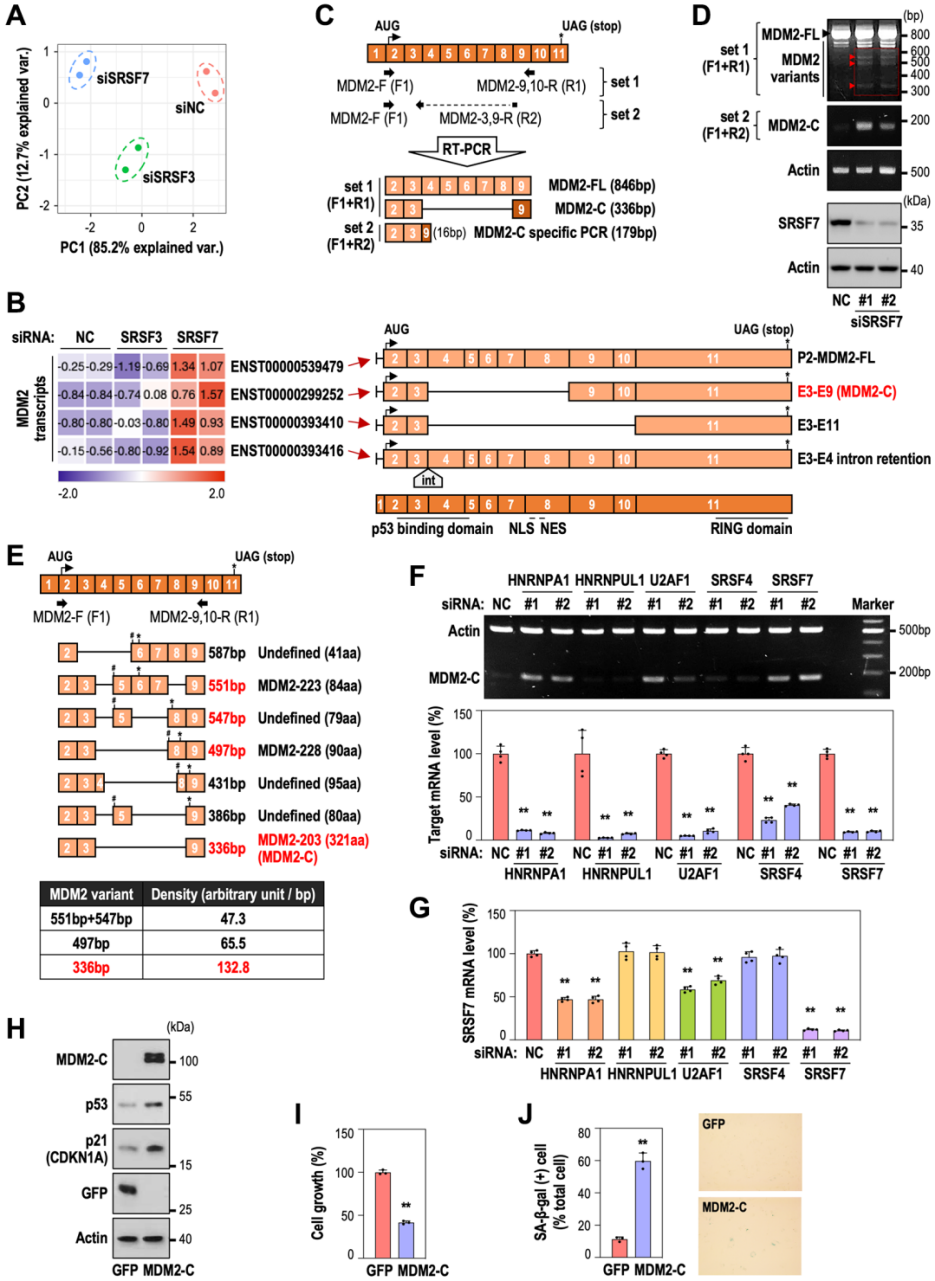
**SRSF7 depletion modulates MDM2-C expression via alternative splicing.** (**A**, **B**) RNA seq analysis. HDFs (DT2) were transfected with siRNA against either NC or SRSF3 or SRSF7 for 3 days. (**A**) Principal component analysis. (**B**) A heatmap of MDM2 transcripts, along with the schematic of each transcript. The values are scaled into z-score. Abbreviations: int: intron; NLS: nuclear localization signal; NES: nuclear export signal. (**C**) A diagram of exon composition of MDM2-FL with primer positions and exon numbers according to the updated MDM2 gene information (NM_002392.6), along with diagrams of predicted PCR products. (**D**, **E**) HDFs (DT2) were transfected with siRNA against either negative control (NC) or SRSF7 for 4 days. (**D**) RT-PCR and western blot analysis. (**E**) Diagrams of MDM2 splice variants identified by DNA sequencing, along with a table showing density per base pair of each variant. (^#^ a point of frameshift occurrence; ^*^ the position of stop codon). (**F**, **G**) HDFs (DT2) were transfected with siRNA against either negative control (NC) or each splicing factor for 3 days. (**F**) RT-PCR analysis of MDM2-C generation, along with mRNA level of each splicing factor using qPCR. (^**^p < 0.01 vs. NC by student *t*-test). (**G**) mRNA level of SRSF7 using qPCR. (^**^p < 0.01 vs. NC by student *t*-test). (**H**–**J**) HDFs (DT2) were infected by the lentiviruses expressing MDM2-C for 3 days. (**H**) Western blot analysis. (**I**) The quantification of cell growth activity (^**^p < 0.01 vs. GFP by student *t*-test). (**J**) The quantification of SA-β-gal (+) cells along with pictures of stained cells. (^**^p < 0.01 vs. GFP by student *t*-test).

To identify which MDM2 splice variant is involved in SRSF7 depletion-mediated senescence, we performed RNA-seq analyses after SRSF7 knockdown. The experimental system was validated by principal component plot, comparing with SRSF3 knockdown which was used as a negative control. (Figure 4A). Expression levels of SRSF7, SRSF3, MDM2 and TP53 were also confirmed as shown in Supplementary Figure 1A. Next, we examined MDM2 transcript expression pattern in each group (Supplementary Figure 1B). We identified four kinds of alternatively spliced MDM2 transcripts which were up-regulated only in SRSF7-depleted samples (Supplementary Figure 1B and Figure 4B). One was MDM2-FL transcript (ENST00000539479), two of them showed exon skipping (ENST00000299252 and ENST00000393410) and the last one included intron retention between exon 3 and 4 (ENST00000393416), adding 31 amino acids without frame shift (Figure 4B). In ENST00000299252 and ENST00000393410 transcripts, exon 4–8 and exon 4–10 were skipped, respectively. Interestingly, both transcripts lack p53 binding domain but retain RING domain, which suggest that they can bind to MDM2-FL and inhibit its function in p53 ubiquitination. Given that ENST00000299252 transcript, which is a known MDM2-C isoform [31], is less spliced out, it could be assumed to represent the function of the two variants with deletion. So, we examined its role in cellular senescence.

To validate the formation of the ENST00000299252 transcript *in vitro*, we designed two primer sets which could detect total MDM2 transcripts (F1+R1) or only MDM2-C isoform using a primer for exon 3-9 junction site (F1+R2) (Figure 4C). In SRSF7-depleted HDF, several MDM2 splice variants were detected by using primer set 1 and MDM2-C specific band was selectively detected by primer set 2 (Figure 4D). Next, we eluted cDNA fragments of the bands of MDM2 variants from the agarose gel (indicated by red rectangle in Figure 4D), subcloned into pGEM^®^-T Vector and performed DNA sequencing to identify the variants. Total seven splice variants were identified (Supplementary Figure 2) and the schematics of each variant are shown in Figure 4E. Three bands observed clearly on the agarose gel (Figure 4D) were probably a mixed one for 551 bp and 547 bp, one for 497 bp, and one for 336 bp (indicated by red triangle and red letters) (Figure 4D, 4E). The density per base pair of each band showed that the 336 bp fragment, which is MDM2-C isoform, was expressed in the highest level (Figure 4E, lower panel). Further, the other six isoforms except 336 bp (MDM2-C) have frame shift derived by exon skipping, respectively at different position, thereby terminating the translation at the earlier codon. These results suggest that MDM2-C isoform may be the major variant, generated by SRSF7 down-regulation, having the functional role unlike the other isoforms. Next, to examine if MDM2-C generation could occur specifically by SRSF7 depletion, HDFs were transfected with siRNA against several splicing regulators, HNRNPA1, HNRNPUL1, U2AF1, SRSF4 and SRSF7. MDM2-C generation was most pronounced in SRSF7-depleted samples (Figure 4F). However, we found that the depletion of HNRNPA1 or U2AF1 also caused MDM2-C formation. To elucidate the link between the splicing regulators and SRSF7, we examined SRSF7 mRNA level after knockdown of the factors. Interestingly, SRSF7 mRNA level was simultaneously decreased in HNRNPA1 or U2AF1-depleted samples (Figure 4G), indicating that the expression of these two splicing factors may be closely related. Finally, we tested the role of MDM2-C in senescence. MDM2-C overexpression in young HDF (DT2) clearly induced senescence, evidenced by p53 and p21 induction (Figure 4H), cell growth inhibition (Figure 4I) and significant gain of SA-β-gal (Figure 4J). These results indicate that SRSF7 suppression generates MDM2-C formation via exon deletion of MDM2, which contributes to p53 accumulation and cellular senescence. Taken together, our results explain that deregulation of splicing modulators occurs at the early period of senescence and SRSF7 down-regulation is a key event to induce cellular senescence via formation of MDM2-C variant and subsequent p53 stabilization.

## DISCUSSION

Alternative splicing is one of the major post-transcriptional processes, and determines which protein isoform could be generated and expressed in cells. The disruption in the regulation of AS can contribute to the altered isoform generation [3], consequently affecting cell physiology [4]. The importance of maintaining AS regulation has been emphasized in cellular senescence and aging [6, 7]. Splicing deregulation has been observed in cellular senescence and aging process, often accompanied by down-regulation of splicing factors [13, 15]. The resulting isoform variation can impact senescence and aging phenotype. Several key splice variants have been identified to be significantly associated with senescence and aging phenotype [16, 21]. Down-regulation of a splicing factor leads to the generation of senescence-associated splice variants and the induction of senescence. However, such isoform generation does not always occur in every senescence process. Cellular senescence is a heterogenous process accompanying various cellular events, thereby involving different molecular mechanisms depending on cell lines and senescence types. Thus, it is not enough to identify one splice isoform in one type of senescence of a specific cell line. Nevertheless, identifying senescence-associated splice variants and their upstream splicing regulators will help to advance our understanding of the impact of splicing alterations in senescence.

It has been unclear what precedes between splicing deregulation and senescence process. Nevertheless, there is increasing evidence that splicing alterations directly contribute to cellular senescence and aging, primarily through the deregulation of splicing factors [7]. Global spliceosome activity has been shown to undergo deregulation, leading to the onset of senescence during conventional replicative senescence [13]. However, it remains to be elucidated what causes the deregulation of splicing factors for the onset of senescence. Alterations in the expression of splicing factors may be caused by changes in the activity of transcription factors [7]. Of importance, such alterations are likely to impose a considerable change in transcriptome of aging cells, generating diverse splice variants which would not be generated in the presence of those splicing factors. Growing number of studies has identified that splice variants derived by altered splicing factors can directly contribute to cellular senescence [7]. Indeed, deregulated splicing factors lead to the formation of splice variants, including senescence-associated splice variants [16, 21]. Those splice variants which would not be generated in the normal states could alter the cell physiology and induce cellular senescence. Further, those senescence-associated splice variants could contribute to the continuous deregulation of splicing factors, consequently helping to establish cellular senescence.

In this study, we aimed at investigating the role of SRSF7, which was found to be consistently down-regulated during RS of human fibroblasts, through the integrative RNA-seq analyses. We observed its down-expression in HDFs during RS and OSIS, and further detected the increased generation of MDM2 splice variants. One of the MDM2 splice variants was MDM2-C, which lost exon 4-8 by AS, and its generation was mainly specific to SRSF7 depletion. Further validation by MDM2-C overexpression was enough to show that MDM2-C could induce cellular senescence by stabilizing p53. The most well-known MDM2 splice variants are MDM2-A, MDM2-B and MDM2-C, and their presence in cells is all relevant to p53 accumulation [35, 37, 38]. MDM2-A and MDM2-B have been shown to be involved in senescence induction by p53 stabilization [37, 38]. MDM2-C has been also reported to contribute to p53 accumulation [35] but not to senescence. Here, we proved that MDM2-C could induce senescence by stabilizing p53 and subsequent induction of its downstream effector, p21. Notably, these three MDM2 splice variants commonly lack p53 binding domain but retain RING domain, suggesting that they can interact with MDM2-FL and inhibit its function in p53 degradation. Here, we demonstrated that MDM2-C presence was closely linked to p53 accumulation and senescence induction, but we did not validate if MDM2-C interacted with MDM2-FL to inhibit its function. Nevertheless, the increased p53 stability in the presence of MDM2-FL implies that MDM2-FL function could be inhibited by MDM2-C. The factors driving SRSF7 downregulation remain unclear. What is clear, however, is that the overexpression of the MDM2-C variant in human diploid fibroblast (DT2) suppresses cell proliferation and induces senescence. In this regard, the regulation of MDM2-C could be considered a cause of cellular senescence.

As RNA binding proteins (RBPs), SRSF proteins serve as trans-acting elements in splicing regulation, mainly recognizing exonic splicing enhancers to promote exon inclusion [18]. Each RBP has its own RNA binding motif in pre-mRNA sequence to interact with [41]. Through RBPmap, a web server that predicts specific binding sites of RBPs on their target RNA sequences [42], we further identified SRSF7 binding motifs in MDM2 pre-mRNA sequence whereby the predicted binding sites reside in exon 4 and exon 8 of MDM2 (Supplementary Table 1). The generation of MDM2-C variant may be attributed to less recognition and, subsequently, skipping of exon 4–8 of MDM2 pre-mRNA in the absence of SRSF7. Further studies on the direct interactions between SRSF7 and MDM2 RNA, and, especially, on the regions of exon 4 and exon 8, need to be validated *in vitro*.

MDM2-C generation in the absence of SRSF7 was clearly validated by MDM2-C specific PCR with a primer that links the junction of exon 3 and 9. However, MDM2-C specific band was also detected when either HNRNPA1 or U2AF1 was depleted. Interestingly, other than the suppression of HNRNPUL1 or SRSF4, where MDM2-C was not detected, we observed co-downregulation of SRSF7 when either HNRNPA1 or U2AF1 was suppressed. No study has yet examined such direct mechanism which would cause SRSF7 down-regulation in the case of HNRNPA1 or U2AF1 depletion. As splicing deregulation accompanied by down-expression of splicing factors is known to be related to senescence induction, SRSF7 down-regulation may be derived from induced senescence with the suppression of these two identified splicing factors, consequently contributing to MDM2-C generation. The relationship between those splicing factors needs to be further elucidated to understand their coordination in cellular senescence. Taken together, the precise splicing regulation accompanied by the balanced expression of splicing factors is critical to maintain cell homeostasis and inhibit senescence. Further assessment of the relationship between deregulation of a specific splicing factor and the altered AS of its target gene could help to identify the role of senescence-associated splice isoforms in regulating senescence and comprehensively understand global splicing regulation process during senescence.

## MATERIALS AND METHODS

### RNA seq analysis of replicative senescence model

Replicative senescence datasets of human fibroblasts, Wi-38, MRC-5, and BJ, were downloaded from NCBI Gene Expression Omnibus (GEO) under the accession code GSE130306, GSE64553, and GSE63577, respectively. Raw data (FASTQ files) was imported into AltAnalyze software (v2.1.4), which uses the embedded software Kallisto and Ensemble72 annotations. Reads per kilobase of transcript per million mapped reads values and log2 fold change values were obtained and used for analysis. For normalization of gene expression level, z-score was calculated using the built-in R scale function (v4.1.2). Percent Spliced In splicing method called MultiPath-PSI was performed by AltAnalyze to assess differential splicing events during cellular senescence. To assess the enrichment of gene sets of interest, we performed gene set enrichment analysis using the R fgsea package (v1.20.0) [43] based on the Molecular Signatures Database (MSigDB database v7.5.1). Normalized enrichment score and FDR for each gene set were obtained. Morpheus (https://software.broadinstitute.org/morpheus/) was used to generate gene expression heatmaps.

### Transcriptomic analysis of RS and OSIS from microarray data

Previously reported transcriptome data derived from senescence models (RS and OSIS) of HDFs [39, 40] were downloaded from NCBI GEO under the accession codes GSE41714 and GSE80322, respectively. For normalization of transcript expression level, z-score was calculated using the built-in R scale function (v4.1.2). Morpheus (https://software.broadinstitute.org/morpheus/) was used to generate transcriptome expression heatmaps.

### RNA seq analysis of splicing factor knockdown HDFs

Total RNA was purified using NucleoSpin RNA Plus kit (MACHEREY-NAGEL, Düren, Germany). Library preparation, sequencing, and data analysis were performed by Theragen Etex (Seoul, Korea). RNA quality was assessed by analysis of 18S and 28S rRNA band integrity on RNA 6000 Nano Kit using an Agilent Bioanalyzer (Agilent, Santa Clara, CA, USA). Libraries were prepared using the TruSeq Stranded Total RNA Kit with Ribo-Zero Gold (Illumina, San Diego, CA, USA). The cDNA library size and quality were evaluated electrophoretically with an Agilent DNA 1000 Kit (part # 5067-1504) (Agilent, Santa Clara, CA, USA) and the final product should be a band at approximately 260 bp. Subsequently, the libraries were sequenced using Illumina HiSeq2500 that were set to rapid run mode. Raw data (FASTQ files) was imported into AltAnalyze software (v2.1.4). Reads per kilobase of transcript per million mapped reads values and log2 fold change values were obtained and used for analysis. For normalization of gene and transcript expression level, z-score was calculated using the built-in R scale function (v4.1.2). Principal component analysis was performed using the prcomp function in the R stats package (v4.1.2). Morpheus (https://software.broadinstitute.org/morpheus/) was used to generate expression heatmaps at the genomic and transcriptomic levels.

### Cell culture, cell growth and development of cellular senescence

Primary HDFs were provided by Dr. Lim IK (Ajou University) [44] and were cultured in DMEM supplemented with 10% FBS (Gibco, Grand Island, NY, USA) and antibiotics (Antibiotic-Antimycotic, Gibco) at 37°C in a humidified incubator with 5% CO_2_ as previously described [39]. Cell growth and viability were monitored by the trypan blue staining. At the end point of each experiment, cells were harvested by trypsinization and counted using the countess automated cell counter (Invitrogen, Carlsbad, CA, USA) after staining with 0.4% (w/v) trypan blue (Invitrogen) to exclude the dead cells.

To develop RS, confluent HDFs were continuously subcultured by being transferred evenly into two new dishes, and the numbers of population doublings (PDs) as well as the doubling times (DTs) were continuously monitored as described previously [39]. To generate OSIS of HDFs, primary HDFs of DT2 were exposed to H_2_O_2_ as previously described [40].

### SA-β-gal activity staining

Cells seeded on twelve-well plates were subjected to SA-β-gal activity staining. Gain of SA-β-gal activity was monitored by using the SA-β-gal staining kit (CST, #9860S, Danvers, MA, USA) according to the instruction provided. The stain was visible 12 hours after incubation at 37°C. Representative images from each well were taken using Leica DMi1 inverted microscope (Leica Microsystems, Wetzlar, Germany). By counting the numbers of the blue-stained and total cells using Image J software (National Institutes of Health, https://imagej.nih.gov/ij/), the percentage of SA-β-gal positive cells was obtained.

### Transfection of siRNAs into cells

HDFs were transfected with siRNA duplexes for the target genes using Lipofectamine RNAiMAX^™^ reagent (Invitrogen, Carlsbad, CA, USA) according to the instruction provided. Target siRNA duplexes were generated by Bioneer (Daejeon, Korea) and their sequences are as follows: SRSF7 (#1, 5′-GUCUGU AAAGUGUAACCUA; #2, 5′-GGAUCGAGGUA UUUCCAAU), MDM2 (#1, 5′-CUUAUUCUCCA GCCUCUUU; #2, 5′-CAUUGAACCUUG UGUGAUU), HNRNPA1 (#1, 5′-CAACUUCGGUCG UGGAGGA; #2, 5′-UCCACGACCACCACCAAAG), HNRNPUL1 (#1, 5′-AGUUUGCAGAGAACGAUGU; #2, 5′-CAGAUCAUGCGGUCUUAGA), U2AF1 (#1, 5′-GCUAGAAAGUGUUGUAGUU; #2, 5′-GCCAU GCCAUUUUUACCUU), SRSF4 (#1, 5′-CACAAGU GAUUGGAGUAGA; #2, 5′-CAGAGUACA GACUUAUUGU) and negative control (5′-CCU ACGCCACCAAUUUGGU).

### Production of MDM2-C overexpressing lentiviruses

The MDM2-C overexpression plasmid (pCMV6-MDM2-C-AC-GFP) was purchased from OriGene Technologies (Rockville, MD, USA) and MDM2-C CDS was serially subcloned into Sgf1 and Mlu1 sites of pLenti-C-mGFP plasmid (OriGene Technologies, Rockville, MD, USA) to generate pLenti-MDM2-C-GFP plasmid. To produce the recombinant lentivirus harboring MDM2-C, a 293T packaging cell was transfected with pLenti-MDM2-C-GFP plasmid using Lipofectamine2000 (Invitrogen). Medium containing recombinant lentivirus was harvested 2 days after transfection and filtered through a 0.45 μm filter (Pall Life Sciences). A filtered medium was mixed with polybrene (8 μg/mL, Sigma-Aldrich) for facilitated infection. After the infection with the recombinant lentivirus, cells were incubated for 12 hours to be changed to the fresh medium and harvested 3 days after the infection.

### Western blot analysis

Cells were washed twice with PBS and lysed in RIPA lysis buffer (150 mM NaCl, 1% NP-40, 0.5% Sodium deoxycholate, 0.1% SDS, 50 mM Tris (pH 8.0), 1 mM NaF, 1 mM Na_3_VO_4_, 1 mM PMSF, 1.5 μg/ml pepstatin A, 1.5 μg/ml leupeptin). Antibody for SRSF7 (ab138022) was purchased from Abcam (Cambridge, MA, USA), and antibody for turboGFP (TA150041) was obtained from OriGene (Rockville, MD, USA). Antibodies for p21 (sc-6246), p53 (sc-126), MDM2 (sc-56154) and β-Actin (sc-8432) were purchased from Santa Cruz (Dallas, TX, USA).

### Detection of splice variants by RT-PCR and DNA sequencing

Total RNA was isolated using TRIzol (Invitrogen) and cDNA was prepared using ReverTra Ace^™^ qPCR RT Kit (Toyobo Co. Ltd., Osaka, Japan). PCR was performed using Ex-taq (Takara, Kyoto, Japan) with 35 cycles of the reaction and the reaction were as follows: total MDM2 (F1+R1: 95°C for 30 seconds, 54°C for 30 seconds, and 72°C for 1 minute), MDM2-C (F1+R2: 95°C for 30 seconds, 58°C for 30 seconds, and 72°C for 1 minute), Actin (95°C for 30 seconds, 58°C for 30 seconds, and 72°C for 1 minute). PCR product was applied to agarose (1.5%) gel electrophoresis and visualized by SL-20 DNA Image Visualizer (Seoulin Scientific, Gyeonggi, Korea) with Midori Green Advance (Nippon Genetics, Düren, Germany). The bands of MDM2 splice variants were eluted and inserted into pGEM^®^-T Vector (Promega, Fitchburg, WI, USA) for TA cloning. The clones including an insert band were selected, and their splicing patterns were confirmed by DNA sequencing performed by Cosmo Genetech (Seoul, Korea). The PCR primer sets for MDM2 and Actin were produced by Cosmo Genetech (Seoul, Korea) as follows: MDM2-F (F1), 5′-CGCGAAAACCCCGGATGGTG; MDM2-9,10-R (R1), 5′-ACAGTAACTTGATATACCTCATC; MDM2-3,9-R (R2), 5′-CACCAGCATCAAGATCCTC; Actin-F, 5′-GCACTCTTCCAGCCTTCCTT; Actin-R, 5′-CTGTCACCTTCACCGTTCCA.

### Quantitative real-time PCR (qPCR) for mRNA expression

Total cellular RNAs were isolated using TRIzol (Invitrogen), and their cDNAs were prepared using ReverTra Ace^™^ qPCR RT Kit (Toyobo Co. Ltd., Osaka, Japan). PCR was performed using GoTaq^®^ qPCR Master Mix (Promega, Fitchburg, WI, USA) according to the protocol provided. The primer sets were produced by Cosmo Genetech (Seoul, Korea) as follows: SRSF7, 5′-AAGAAGCAGCCGATCAAAG and 5′-AAAGGG TGAACTTGAGAGC; TP53, 5′-CTCACCATCAT CACACTGGAA and 5′-TCATTCAGCTCTCGG AACATC; CDKN1A, 5′-TCACTGTCTTGTACCCTT GTGC and 5′-GGCGTTTGGAGTGGTAGAAA; MDM2, 5′-CAGGCAAATGTGCAATACCAAC and 5′-GCTTTGGTCTAACCAGG; HNRNPA1, 5′-AT GGGAGAATGCACACGAGG and 5′-CTCA TTACCACACAGTCCGTG; HNRNPUL1, 5′-CATC AACGAGGAGGTCGAGA and 5′-CATGGCAT AATGTCCGTCCG; U2AF1, 5′-ATGGGAGAA TGCACACGAGG and 5′-TCTATGCTTCTT GCGACGGC; SRSF4, 5′-TCATTCAAGG TCTCGCTCTCG and 5′-ACCTGGACCGAG ATCTACTCT; TBP, 5′-CACCTTACGCTCAGGGCTT and 5′-CTGAATAGGCTGTGGGGTCA.

### Statistical analyses

Statistical analyses of transcriptomes were performed using R (v4.1.2) as described above, and that of the other experimental data was evaluated by unpaired two-samples Student’s *t*-test using GraphPad Prism software (La Jolla, CA, USA).

### Data availability

Bulk RNA seq and microarray datasets analyzed in this study can be found at the NCBI GEO under the accession codes GSE130306, GSE64553, GSE63577, GSE41714, GSE80322 and GSE230443.

## Supplementary Materials

Supplementary Figures

Supplementary Table 1
