# Supplementary material for: SRSF7 downregulation induces cellular senescence through generation of MDM2 variants
Source: Aging (Albany NY). 2023 Dec 29;15(24):14591–606. doi: 10.18632/aging.205420 (PMC10781460; doi:10.18632/aging.205420)
Supplement: Supplementary Figures [file aging-15-205420-s001.pdf]

# SUPPLEMENTARY FIGURES

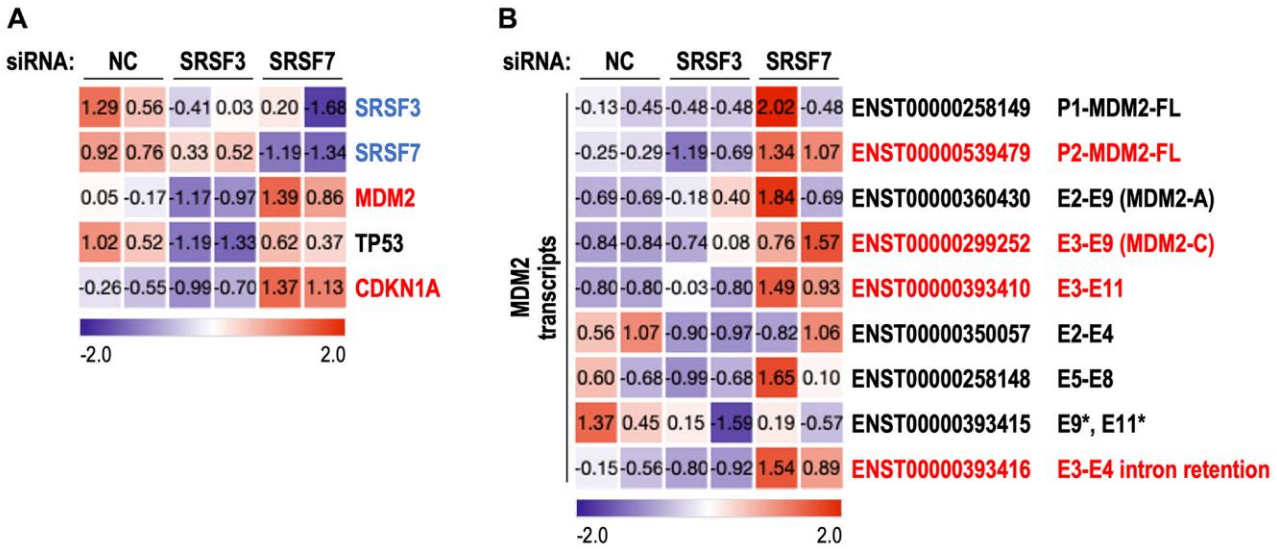


**Supplementary Figure 1. Gene and transcript expression heatmaps of HDFs transfected with siRNA against either SRSF3 or SRSF7.** The values are scaled into z-score. (**A**) Gene expression heatmap. (**B**) Transcript expression heatmap of MDM2 splice variants. EMSEMBLE ID and splicing information are displayed together. Transcripts increased exclusively in SRSF7 knockdown samples are expressed in red color.

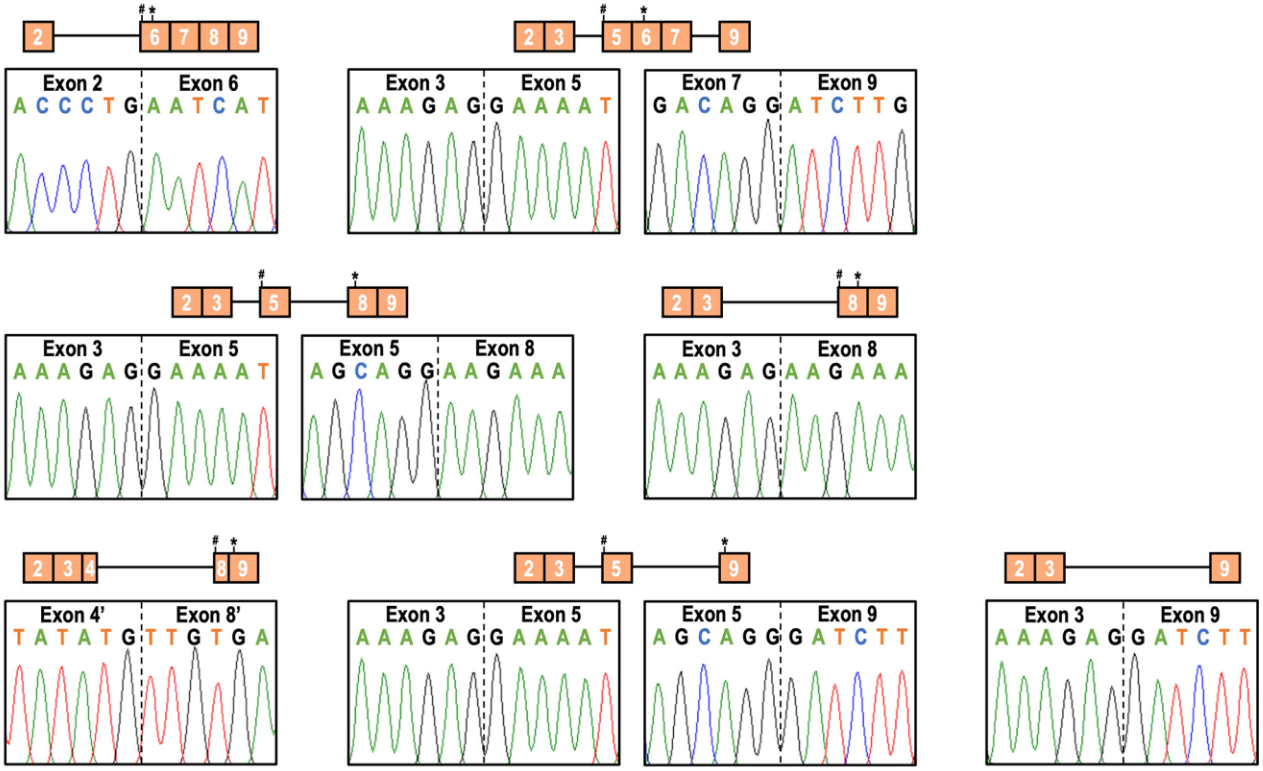


**Supplementary Figure 2. The schematics of DNA sequencing results of MDM2 splice variants generated by SRSF7 knockdown.** Each variant is displayed by a schematic of exons included in the transcript and the graph of DNA sequencing result indicates the exon junctions where exon skipping occurred. (# a point of frameshift occurrence; * the position of stop codon).
