# Supplementary material for: SRSF7 downregulation induces cellular senescence through generation of MDM2 variants
Source: Aging (Albany NY). 2023 Dec 29;15(24):14591–606. doi: 10.18632/aging.205420 (PMC10781460; doi:10.18632/aging.205420)
Supplement: Supplementary Table 1 [file aging-15-205420-s002.pdf]

SUPPLEMENTARY TABLE

Supplementary Table 1. RBPmap results for the prediction of SRSF7 binding sites on MDM2 RNA.

Position	Exon	Motif	K-mer	Z-score	*P*-value
83	1	acgacg	gccgag	1.897	2.89E-02
96	1	acgacg	ggcggc	1.897	2.89E-02
102	1	acgacg	cgcgac	2.192	1.42E-02
510	4	acgacg	aacgau	2.244	1.24E-02
852	8	acgacg	aacgac	2.256	1.20E-02
864	8	acgacg	aacgcc	1.731	4.17E-02
4197	11	acgacg	aaggac	2.423	7.70E-03
4200	11	acgacg	gacuac	2.603	4.62E-03
4214	11	acgacg	caggac	2.359	9.16E-03
